# A Nutraceutical Rich in Docosahexaenoic Acid Improves Portal Hypertension in a Preclinical Model of Advanced Chronic Liver Disease

**DOI:** 10.3390/nu11102358

**Published:** 2019-10-03

**Authors:** Zoe Boyer-Diaz, Joan Carles Domingo, Estefanía De Gregorio, Nicolò Manicardi, Peio Aristu-Zabalza, Begoña Cordobilla, Laia Abad-Jordà, Martí Ortega-Ribera, Anabel Fernández-Iglesias, Montserrat Marí, Jaime Bosch, Jordi Gracia-Sancho

**Affiliations:** 1 Barcelona Liver Bioservices, 08036 Barcelona, Spain; 2 Biochemistry and Molecular Biomedicine Department, Faculty of Biology, University of Barcelona, 08036 Barcelona, Spain; 3 Department of Cell Death and Proliferation, IIBB-CSIC/IDIBAPS, 08036 Barcelona, Spain; 4 Liver Vascular Biology Research Group, IDIBAPS, 08036 Barcelona, Spain; 5 CIBEREHD, 28029 Madrid, Spain; 6 Hepatology, Department of Biomedical Research, University of Bern, 3012 Bern, Switzerland

**Keywords:** DHA, omega-3, liver fibrosis, liver cirrhosis, hepatic hemodynamics

## Abstract

Inflammation and oxidative stress play a key role in the pathophysiology of advanced chronic liver disease (ACLD) and portal hypertension (PH). Considering the current lack of effective treatments, we evaluated an anti-inflammatory and antioxidant nutraceutical rich in docosahexaenoic acid (DHA) as a possible therapy for ACLD. We investigated the effects of two-week DHA supplementation (500 mg/kg) on hepatic fatty acids, PH, oxidative stress, inflammation, and hepatic stellate cell (HSC) phenotype in rats with ACLD. Additionally, the effects of DHA were evaluated in murine macrophages and human HSC. In contrast to vehicle-treated animals, cirrhotic rats receiving DHA reestablished a healthy hepatic fatty acid profile, which was associated with an improvement in PH. The mechanisms underlying this hemodynamic improvement included a reduction in oxidative stress and inflammation, as well as a marked HSC deactivation, confirmed in human HSC. Experiments with cultured macrophages showed that treatment with DHA protects against pro-inflammatory insults. The present preclinical study demonstrates that a nutraceutical rich in DHA significantly improves PH in chronic liver disease mainly by suppressing inflammation and oxidative stress-driven HSC activation, encouraging its evaluation as a new treatment for PH and cirrhosis.

## 1. Introduction

Advanced chronic liver disease (ACLD) constitutes an important clinical problem worldwide, causing 1.03 million deaths per year [1]. Unfortunately, current therapeutic options to treat ACLD are limited, with a lack of effective and safe treatments targeting the main mechanisms mediating the disease and its complications [2,3].

Alcohol abuse, viral infection, and metabolic factors are some of the main causes of ACLD. Continuous exposure to these harmful agents causes liver cells to deteriorate and die, releasing signals which, in turn, lead to severe hepatic inflammation and oxidative stress. In this environment, hepatic stellate cells (HSC) shift to their activated state and begin an abnormally high deposition of extracellular matrix, causing liver fibrosis [4,5]. In addition, liver sinusoidal endothelial cells (LSEC) undergo a capillarization process, losing their characteristic fenestrae and developing a basal membrane, which interferes with the communication between sinusoidal blood and parenchymal cells [6]. Altogether, the ensuing parenchymal and sinusoidal dysfunction contributes to the development of portal hypertension (PH). PH is considered ACLD’s main complication and becomes clinically significant (associated with the risk of potentially lethal clinical complications) when the hepatic venous pressure gradient increases over 10 mmHg. At advanced stages, PH can ultimately lead to bleeding of esophageal varices, ascites and kidney failure, hepatic encephalopathy, among other serious complications [7].

Previous studies have shown that ω-3 and ω-6 polyunsaturated fatty acid (PUFA) levels are deregulated during liver disease [8,9,10]. However, the role of hepatic fatty acids, which may have severe effects on hepatocyte viability, inflammation, and survival, in the context of ACLD is still poorly understood. Docosahexaenoic acid (DHA) is an ω-3 essential fatty acid that cannot be synthesized by the human body and must be obtained from the diet. It is known that ω-3 PUFAs, and DHA in particular, possess strong anti-inflammatory and antioxidant properties. Its pleiotropic effects are complex and include the activation of antioxidant pathways, inflammation resolution by pro-resolving lipid mediators such as resolvins and protectins, increased leukocyte migration, inhibition of pro-inflammatory cytokines and eicosanoid synthesis, decreased lipid peroxidation, and regulation of key transcription factors such as nuclear factor kappa B (NFκB) and peroxisome proliferator-activated receptor gamma (PPAR-γ), among others [11,12]; altogether suggesting that DHA supplementation could have beneficial effects in ACLD.

In this study, we aimed, in the first place, at characterizing the hepatic fatty acid deregulations that may take place in ACLD. Given the possible nature of these alterations, and taking into account the anti-inflammatory and antioxidant properties of ω-3 PUFAs, we then proposed a nutraceutical rich in DHA triglycerides as a possible approach to reestablish a healthy lipid profile and evaluated its effects on ACLD and PH.

## 2. Materials and Methods 

### 2.1. Animal Model of Advanced Chronic Liver Disease

Advanced chronic liver disease (ACLD) was induced in male Sprague–Dawley rats (150 g, Janvier) by administration of thioacetamide (TAA, Sigma) twice per week during a total of 12 weeks. For that purpose, TAA was dissolved in saline solution at 125 mg/mL and administered intraperitoneally at a dose of 250 mg/kg body weight, as previously described [13,14]. Healthy male Sprague–Dawley rats with a similar final body weight (300 g, Janvier) were used as a control.

Animals were maintained in an environmentally controlled animal facility. All procedures were approved by the Laboratory Animal Care and Use Committee of the University of Barcelona and were conducted in accordance with the European Community guidelines for the protection of animals used for experimental and other scientific purposes (EEC Directive 86/609).

### 2.2. Docosahexaenoic Acid Administration

After the last TAA administration, animals with ACLD underwent a detoxification period of 2–5 days before beginning pharmacological treatment. A total of six healthy rats and 15 cirrhotic rats were enrolled per group and randomized to receive fish oil enriched in docosahexaenoic acid (DHA, 500 mg/kg body weight/day, Brudy Technology, patent W2007/071733A3) or a standard vehicle, used as placebo (1% methylcellulose + 0.05% poloxamer). Both treatments were administered by gavage, daily, for a total of two weeks. DHA or vehicle was administered by a third person, and therefore, the investigators administering the drug and performing the experiments were not aware of the treatment received by the rats. This blinding was maintained until the final analysis of results. No treatment-related mortality was registered. However, due to technical issues during the subsequent in vivo hemodynamic studies, the final number of animals per group was n = 6 for healthy rats receiving vehicle, n = 6 for healthy rats receiving DHA, n = 11 for cirrhotic rats receiving vehicle, and n = 14 for cirrhotic rats receiving DHA.

### 2.3. In Vivo Hemodynamic Analysis

To avoid alterations in splanchnic hemodynamics, animals were food-deprived for 8–12 h previous to the study. One hour after the last DHA dosage, rats were anesthetized with ketamine hydrochloride (100 mg/kg body weight; Imalgene 1000; Merial Laboratories) plus midazolam (5 mg/kg body weight; Laboratorio Reig Jofré), administered intraperitoneally.

To facilitate breathing during anesthesia, a tracheotomy was performed with a p240 catheter (Portex). Mean arterial pressure (MAP) and heart rate (HR) were measured by cannulating the femoral artery, and portal pressure (PP) was measured by cannulating the ileocolic vein, both with a heparinized p50 catheter (Portex) connected to a pressure probe. Portal blood flow (PBF) and superior mesenteric arterial blood flow (SMABF) were determined with the help of specific non-constrictive perivascular ultrasonic transit-time flow probes (Transonic Systems Inc., Ithaca, NY, USA). In order to avoid portal-collateral blood flow, PBF was measured after the splanchnic vein bifurcation, next to the entrance to the liver. Pressure and flow probes were connected to a PowerLab (4SP) and data were displayed into a Chart v5.5.6 software file. Hemodynamic parameters were acquired following a 20 min stabilization period [15]. After completion of the hemodynamic study, blood and tissue samples were collected for molecular and histological determinations.

### 2.4. Biochemical Analysis

Fatty acid composition in liver tissue was determined by measuring methyl ester levels following methylation reaction, using the method described by Lepage and Roy [16]. Gas chromatography (GC) analysis was performed using a Shimadzu GCMS-QP2010 Plus gas chromatograph/mass spectrometer (Shimadzu) with a Shimadzu AOC-20i autoinjector and a Shimadzu AOC-20s autosampler. The column used was a Suprawax-280 (Teknokroma). Data were acquired and analyzed with the help of GCMS solution software. The injector was used in splitless mode and MS ionization mode was electron ionization.

Fatty acid methyl esters (FAME) were identified through mass spectra by comparing the elution pattern and relative retention times of FAME with a reference FAME mixture (GLC-744 Nu-Chek Prep. Inc. Elysian, MN, USA). Results were expressed as molar percentage of total fatty acids. The activity of fatty acid-associated enzymes was estimated from the concentration ratio of the enzyme product to its substrate.

Hepatic oxidative stress was determined as superoxide (O_2_^−^) levels in liver tissue. Liver samples were embedded in optimal cutting temperature (OCT) medium (Leica) and sections were incubated with the fluorescent dye dihydroethidium (Molecular Probes), as previously described [17]. Six fields from each slide were randomly selected. Images were obtained with a fluorescence microscope and quantitative analysis was carried out with ImageJ 1.50e software.

Plasma total antioxidant capacity (TAC) was colorimetrically determined with the help of a Synergy™ H1M, Hybrid Multi-Mode Microplate Reader (BioTek Instruments, Inc., Winooski, VT), as previously described [18]. Plasma TAC, expressed as μM copper-reducing equivalent values, was measured using OxiSelect Total Capacity Assay kit (STA-360, Cell Biolabs Inc., San Diego, CA, USA) following manufacturer’s instructions.

Serum aspartate aminotransferase (AST) and albumin levels were determined through standard methods at the Hospital Clinic’s core facilities.

### 2.5. Liver Histology

Liver tissue samples were fixed in 4% formaldehyde (Sigma), embedded in paraffin, sectioned and stained with 0.1% Sirius Red in picric acid aqueous solution (Sigma). Ten images from each slide were analyzed using a microscope (Zeiss Axiovert) equipped with a digital camera and the red-stained area was quantified using AxioVision software (Zeiss Axiovert). Values are represented by mean of fibrosis percentage per total area [19].

### 2.6. Oil Red O Staining

For steatosis assessment, liver tissue samples were embedded in OCT, sectioned, and stained with Oil red O (Sigma). Fifteen images from each slide were analyzed using a microscope (Zeiss Axiovert) equipped with a digital camera. Lipid droplets were evaluated as the percentage of red-stained area per total area, quantified using AxioVision software (Zeiss Axiovert).

### 2.7. Immunofluorescence

Liver tissue samples were fixed in 4% formaldehyde (Sigma), embedded in paraffin, sectioned, and processed for immunofluorescence as previously described [20]. Liver sections were incubated with a primary antibody against CD68 (1:100, MCA341R, Bio-Rad), a secondary Alexa Fluor 555 antibody (1:300, Life technologies), and 40,6-diamino-2-fenilindol (DAPI) (1:1000, Sigma, Kawasaki, Japan) and mounted in Fluoromount G medium. Fifteen images from each slide were analyzed using a fluorescence microscope (Olympus BX-41) and quantitative analysis was carried out with ImageJ 1.50e software. Results are expressed as the percentage of CD68 positive area per total area.

### 2.8. Cell Culture and Docosahexaenoic Acid Treatment

Primary murine peritoneal macrophages (PMs) from C57BL/6 mice were harvested by peritoneal lavage using ice-cold Ca^2+^ and Mg^2+^-free PBS, after three days of treatment with 3% thioglycolate. Murine macrophages from the RAW264.7 cell line and PMs were cultured in Roswell Park Memorial Institute medium (RPMI) supplemented with 10% *v*/*v* fetal bovine serum (Reactiva), 2 mM L-glutamine (Gibco), 100 U/mL penicillin (Reactiva), and 200 μg/mL streptomycin (Reactiva). Both cell types were exposed to serum free medium for 24 h to obtain quiescent cells before being exposed to lipopolysaccharide (LPS). RAW264.7 cells were treated with 10 μM DHA or vehicle (ethanol 0.1%–0.5% *v*/*v*) for 72 h, followed by 50 ng/mL LPS or 0.25 mM palmitic acid (PA) for 4 h. Mouse peritoneal macrophages were treated with 7.5 μM DHA or vehicle for 72 h, followed by 50 ng/mL LPS or 100 ng/mL tumor necrosis factor alpha (TNFα) for 6 h.

LX-2 human hepatic stellate cells (HSC) were seeded onto p6 plates at a density of 60,000 per plate in High-Glucose Dulbecco’s Modified Eagle Medium (DMEM, Biological Industries) supplemented with 10% *v*/*v* fetal bovine serum, 4 mM L-glutamine, 100 U/mL penicillin, and 200 μg/mL streptomycin and cultured for 24 h. Cells were then incubated with increasing concentrations of DHA (10 and 50 μM) or vehicle (ethanol 0.1%–0.5% *v*/*v*) for 72 h.

### 2.9. RNA Isolation and Quantitative PCR

RNA was extracted from cells and liver tissue using RNeasy mini kit (Qiagen, Hilden, Germany) and Trizol (Life Technologies, Carlsbad, CA, USA) respectively, and quantified with the help of a NanoDrop spectrophotometer. Reverse transcription was carried out following QuantiTect reverse transcription kit (Qiagen). qPCR was performed using PowerUp SYBR Green Master Mix (Thermo Fisher, Waltham, MA, USA) and the primers are described in Supplementary Table S1. All amplification reactions were performed as duplicates and nuclease free water was used as a no template control. Results are expressed as 2−ΔΔCt, relative to the endogenous control GAPDH or β-actin.

### 2.10. Western Blot Analysis

Triton lysis buffer was used for protein extraction of both LX-2 cells and liver tissue samples. Nuclear extracts from RAW264.7 cells were isolated as previously described [21]. Proteins were separated by molecular weight by electrophoresis using a sodium dodecylsulfate polyacrylamide gel and transferred to a nitrocellulose membrane. To avoid non-specific binding, membranes were blocked with Tris-buffered saline containing 0.05% Tween-20 and 3% bovine serum albumin or 5% dry milk. Membranes were then incubated overnight with primary antibodies, at 4 °C and 1 h with the corresponding horseradish peroxidase-conjugated secondary antibodies, at room temperature [22]. A complete list of the antibodies used is provided in Supplementary Table S2. Lastly, chemiluminescent signal levels were detected with a LAS4000 (GE Healthcare, Chicago, IL, USA), and protein expression was determined by densitometric analysis using the Image Studio Lite software (LI-COR). Results are expressed as relative densitometric values normalized to endogenous control glyceraldehyde 3-phosphate dehydrogenase (GAPDH) or Lamin A/C.

### 2.11. Statistical Analysis

Results were analyzed by GraphPad Prism v5.0.0 (GraphPad Software, San Diego, CA, USA). Data in the figures are expressed as mean ± standard error of the mean (S.E.M.). Differences among groups were tested for statistical significance by Student’s *t* test, when comparing two groups, or by two-way ANOVA followed by post hoc Tukey’s test, when two different factors were involved. Differences were considered significant at *p* < 0.05.

## 3. Results

### 3.1. Hepatic Fatty Acid Profile Is Deregulated in Rats with ACLD

Rats with ACLD exhibited a profoundly deregulated hepatic fatty acid profile, both structurally and enzymatically. Compared to healthy rats (Table 1), cirrhotic rats presented higher monounsaturated fatty acid (MUFA; +55%) and lower polyunsaturated fatty acid levels (PUFA; −14%). In particular, oleic acid (OA), arachidonic acid (AA), and DHA were notably affected (+187%, −22%, and −66%, respectively). These alterations led to changed cell membrane characteristics (−36% SFA/MUFA and −80% PUFA/MUFA ratios), a shift to pro-inflammatory phenotype (+44% AA/EPA and +141% AA/DHA ratios), and a decrease in ω-3 index (−65%), as shown in Table 2. Moreover, changes in enzymatic activity were observed, with an increase of SCD18 index (C18:1 n-9/C18:0) and a reduction of elongase activities (C18:0/C16:0, C24:0/C22:0 and C24:0/C20:0) (Table 2). Altogether, these data indicate that the pathology increases de novo lipogenesis in the liver and alters FA metabolism, pathways that are recognized as important contributors to the development of metabolic disorders.

### 3.2. Treatment with DHA Reestablishes a Healthy Lipid Profile

When treated with DHA, cirrhotic animals presented a remarkable improvement in their hepatic fatty acid composition, reaching values comparable to those of healthy animals (Table 1). As expected, rats that received DHA showed higher ω-3 and lower ω-6 levels compared to those receiving placebo. Other fatty acid families also presented changes, such as SFA and MUFA (+8% and −37%, respectively), with a striking reduction of OA levels (−43%). Membrane characteristics (+68% SFA/MUFA and +80% PUFA/MUFA ratios) and anti-inflammatory capacity (−94% AA/EPA and −88% AA/DHA ratios) were recovered, as well as a normal fatty acid enzymatic activity (Table 2). Consequently, there was a specific raise in elongase activities and as well as a decrease of SCD18 index.

### 3.3. DHA Ameliorates Portal Hypertension in Rats with ACLD

As shown in Table 3, rats with ACLD receiving DHA exhibited a significant improvement in portal hypertension (−13.37% in portal pressure in vivo) compared to those receiving vehicle. This reduction in portal pressure was not accompanied by a decrease in portal blood flow, thus suggesting an amelioration in intrahepatic vascular resistance (−24.57%). No significant changes were observed in systemic hemodynamic parameters such as mean arterial pressure and heart rate, or in general features such as body, liver, and spleen weight (Supplementary Table S3).

Analysis of serum markers revealed that DHA-treated animals also presented significantly lower levels of serum aspartate aminotransferase (−25.67%), indicating an improvement of hepatic necro-inflammation, whereas albumin levels remained unchanged (Table 3).

### 3.4. DHA Reduces Oxidative Stress and Inflammation

**Figure 1:**
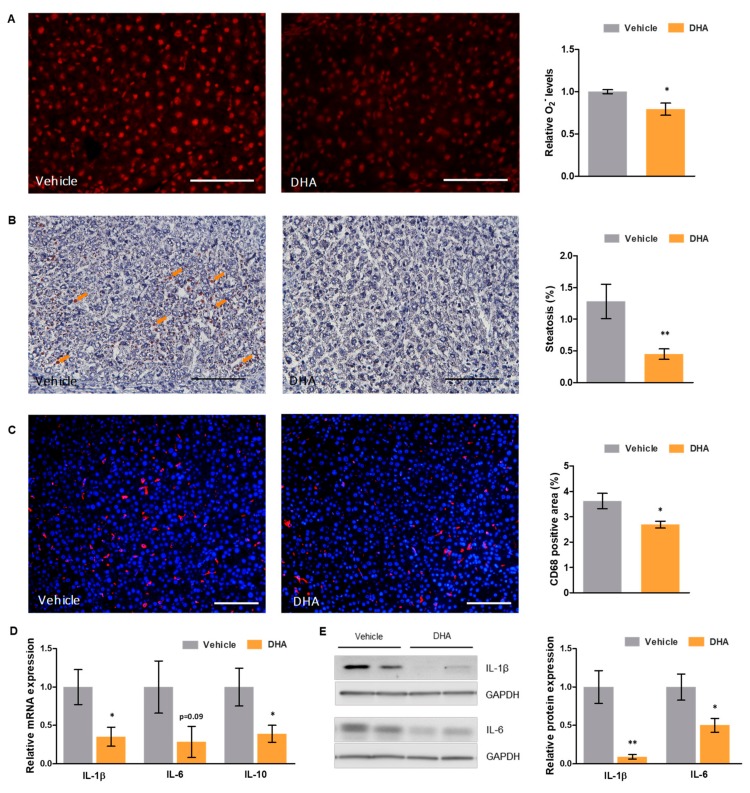
Effects of DHA on oxidative stress and inflammation in a rat model of ACLD. (**A**) Representative images of dihydroethidium staining in liver tissue sections from cirrhotic rats treated with DHA or vehicle (left—scale bar represents 200 μm), and corresponding quantification of superoxide (O_2_^−^) levels (right). (**B**) Representative images of oil red O staining of liver tissue cryosections from cirrhotic rats treated with DHA or vehicle (left—scale bar represents 100 μm), and corresponding quantification (right). (**C**) Representative images of CD68 immunofluorescence in liver tissue sections from cirrhotic rats treated with DHA or vehicle (left—scale bar represents 200 μm; CD68 positive cells in red and nuclei in blue), and corresponding quantification (right). (**D**) Relative mRNA and (**E**) protein expression of key interleukins in total liver tissue from cirrhotic rats treated with DHA or vehicle, normalized to GAPDH. Results are expressed as mean ± S.E.M. * *p* < 0.05 and ** *p* < 0.01 vs. vehicle. *n* = 3 per group (**A**), *n* = 11 per group (**B**, **C**), and *n* = 10 per group (**D**,**E**).

Hepatic and systemic oxidative stress were evaluated as superoxide (O_2_^−^) levels in liver tissue and plasma total antioxidant capacity (TAC), respectively. Rats with ACLD that received DHA presented significantly lower superoxide levels than those that received vehicle (−20.70%; Figure 1A) as well as a higher TAC, although this increase did not reach statistical significance (+24.87%, *p* = 0.1).

Hepatic steatosis, measured as the percentage of oil red O positive area, was significantly decreased in DHA-treated animals, with a 64.99% reduction of lipid droplets compared to those treated with vehicle (Figure 1B).

Moreover, cirrhotic animals treated with DHA showed reduced hepatic inflammation compared to those that received vehicle, with a significantly reduced macrophage infiltration in the liver (−25.69% CD68 positive area; Figure 1C) and the downregulation of key interleukins (IL) that mediate the inflammatory response in ACLD (Figure 1D). Particularly, DHA-treated animals showed significantly reduced IL-1β and IL-10 mRNA expression (−64.68% and −60.97%, respectively), as well as a tendency to decrease IL-6 expression (−71.63%). At the protein level, this downregulation was even greater, with a decrease in expression of 90.99% for IL-1β and 49.85% for IL-6, both statistically significant (Figure 1E).

To evaluate the protective effect of DHA against pro-inflammatory insults, macrophages pre-treated with DHA or vehicle were then supplemented with either LPS, TNFα, or PA, agents known to mediate potent pro-inflammatory responses [23,24].

**Figure 2:**
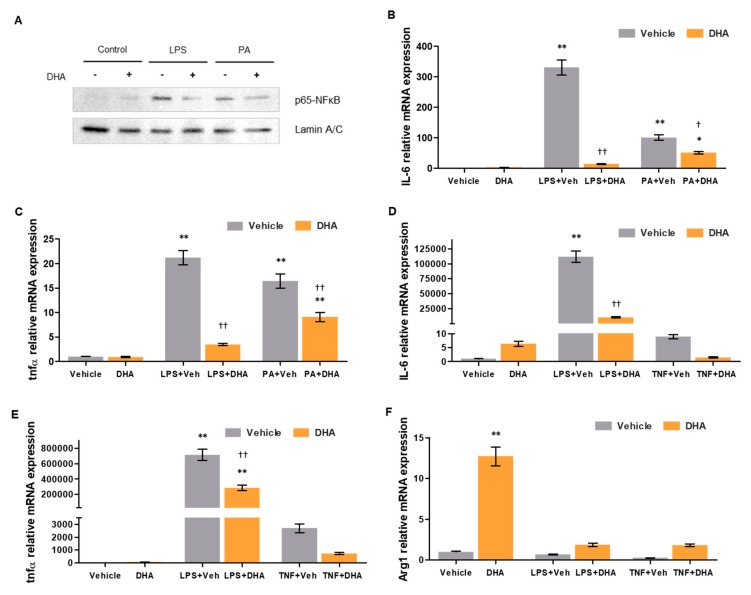
Effects of DHA on macrophage activity. (**A**) p65-NFκB (nuclear factor kappa B) nuclear protein expression in murine macrophages from the RAW264.7 cell line treated with DHA (10 µM) or vehicle and undergoing no challenge, lipopolysaccharide (LPS) challenge or PA challenge. Lamin A/C expression is shown as a loading control. (**B**) Interleukin 6 (IL-6) and (**C**) tumor necrosis factor alpha (TNFα) relative mRNA expression in murine macrophages from the RAW264.7 cell line treated with DHA or vehicle and undergoing no challenge, LPS challenge or palmitic acid (PA) challenge, normalized to β-actin. (**D**) IL-6, (**E**) TNFα and (**F**) Arg1 relative mRNA expression in primary murine peritoneal macrophages treated with DHA (7.5 µM) or vehicle and undergoing no challenge, LPS challenge or TNFα challenge, normalized to β-actin. Results are expressed as mean ± S.E.M. * *p* < 0.05 and ** *p* < 0.01 vs. vehicle without challenge; † *p* < 0.05 and †† p < 0.01 vs. vehicle under the same challenge conditions. *n* = 4 per group (**B**,**C**), and *n* = 3 per group (**D**–**F**).

Murine macrophages from the RAW264.7 cell line treated with DHA presented lower nuclear levels of NFκB transcription factor than those treated with vehicle, after undergoing lipopolysaccharide (LPS) or palmitic acid (PA) challenges for 30 min (Figure 2A). Similarly, as shown in Figure 2B,C, relative mRNA expression of the pro-inflammatory cytokines IL-6 and tumor necrosis factor alpha (TNFα) was significantly decreased in DHA pre-treated macrophages, compared to vehicle-treated cells, after 4 h of incubation with either LPS (−95.86% and −83.59%, respectively) or PA (−49.70% and −44.91%, respectively).

Primary murine peritoneal macrophages (PMs) isolated from C57BL/6 mice were equally treated with DHA or vehicle and submitted to LPS and TNFα challenges. IL-6 and TNFα mRNA expression after incubation with either LPS (−90.49 and −60.11%, respectively) or TNFα (−83.08% and −72.98%, respectively) was strongly reduced in DHA-treated macrophages compared to those receiving vehicle (Figure 2D,E). Contrarily, expression of the anti-inflammatory enzyme arginase was heavily increased in DHA pre-treated cells both at basal levels (+1171%) and after incubation with LPS (+177.83%) or TNFα (+633.63%), as shown in Figure 2F. Taken together, these results indicate that, not only does DHA have anti-inflammatory effects in the liver and systemically, but that it also grants a protective effect against possible future inflammatory insults.

### 3.5. DHA Promotes HSC Deactivation in Rats with ACLD

**Figure 3:**
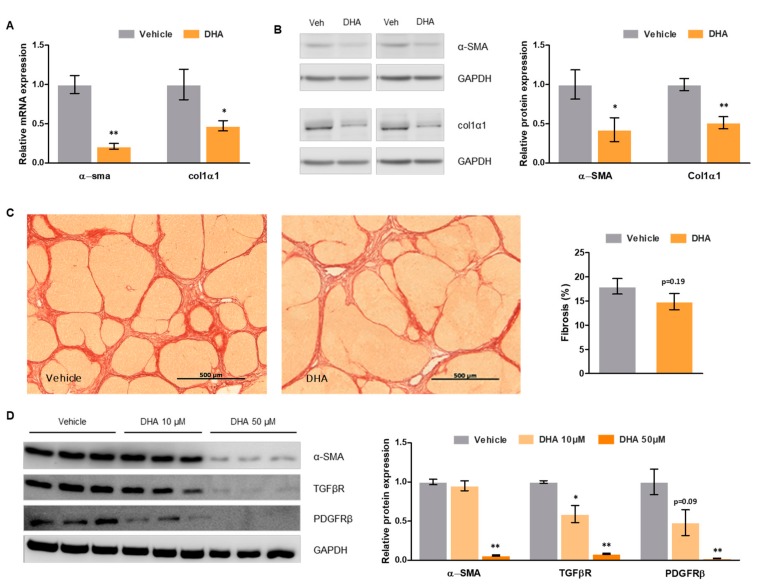
Effects of DHA on hepatic stellate cell (HSC) phenotype and fibrosis. (**A**) α-SMA and collagen-1α1 relative mRNA expression in total liver tissue from cirrhotic rats treated with DHA or vehicle, normalized to GAPDH. (**B**) α-SMA and collagen-1α1 relative protein expression in total liver tissue from cirrhotic rats treated with DHA or vehicle, normalized to GAPDH. (**C**) Representative images of Sirius Red staining of liver tissue sections from cirrhotic rats treated with DHA or vehicle (left—scale bar represents 500 μm), and corresponding quantification (right). (**D**) α-SMA, TGFβR and PDGFβR relative protein expression in LX-2 HSCs treated with DHA (10 and 50 µM) or vehicle, normalized to GAPDH. Results are expressed as mean ± S.E.M. * *p* < 0.05 and ** *p* < 0.01 vs. vehicle. n = 10 per group (**A**–**C**), and *n* = 3 per group (**D**).

In order to evaluate the effects of DHA on HSC phenotype, expression of activation marker alpha smooth muscle actin (α-SMA) was determined (Figure 3A,B). Cirrhotic rats that received DHA presented a striking reduction in α-SMA expression compared to those that received vehicle, both at mRNA (−78.71%) and protein levels (−57.48%), indicating that DHA strongly promotes HSC deactivation. Similarly, collagen-1α1 expression was significantly lower in animals treated with DHA, at mRNA (−48.23%) and protein levels (−48.64%), confirming that HSC deactivation led to a decrease in extracellular matrix generation.

Liver fibrosis, measured as the percentage of Sirius Red positive area, was slightly lower in cirrhotic rats that received DHA than in those receiving vehicle (−18%), although this change was not statistically significant (Figure 3C).

### 3.6. DHA Promotes Deactivation of Human HSC

To assess whether the DHA-mediated HSC deactivation observed in the animal model of ACLD may also occur in the human pathology, human LX-2 HSC were treated with increasing concentrations of DHA. As shown in Figure 3D, DHA promoted HSC deactivation in a concentration-dependent manner, as demonstrated by a reduction in key activation markers such as α-SMA, transforming growth factor beta (TGFβ), and platelet derived growth factor receptor beta (PDGFRβ) (data for 50 μM: −93%, −92%, and −98%, respectively).

## 4. Discussion

Chronic liver injury involves a variety of mechanisms, parts of which are still undefined. In this regard, the role of hepatic fatty acids and their possible implication in hepatocyte viability, inflammation, and survival remains to be elucidated. The present study shows that advanced chronic liver disease (ACLD) pathophysiology involves a profound deregulation in the hepatic fatty acid profile. Said deregulation affects both monounsaturated and polyunsaturated fatty acids, with a marked unbalance in ω-3 and ω-6 essential fatty acids. In particular, docosahexaenoic acid (DHA), known to be a key regulator of many anti-inflammatory and antioxidant processes, presented strikingly low levels in comparison to healthy animals, suggesting possible beneficial effects of DHA supplementation in ACLD. Indeed, treatment with a supplement rich in DHA notably improved hepatic fatty acid composition in cirrhotic animals, reestablishing control levels of many fatty acids and ameliorating membrane quality and anti-inflammatory capacity ratios.

Previous studies had suggested beneficial effects of DHA supplementation in models of acute mild liver injury [25] and non-alcoholic fatty liver disease [26,27,28,29]. In the present study, however, we investigated the effects of DHA in a model of advanced chronic liver disease showing for the first time that it improves portal hypertension. The reduction in portal pressure was not accompanied by a reduction in portal blood flow, pointing to a decrease in intrahepatic vascular resistance as the main mechanism driving this improvement. These results suggest that DHA can have potent effects in improving liver disease, even at very advanced stages, that go beyond reestablishing the liver’s lipid profile.

To determine the underlying mechanisms mediating the improvement of portal hypertension, we evaluated DHA’s effects on oxidative stress, inflammation, hepatic stellate cell (HSC) phenotype, and fibrosis. As expected, given its well-documented antioxidant and anti-inflammatory properties [11,12,30,31], cirrhotic animals receiving DHA presented decreased hepatic oxidative stress and inflammation levels. Moreover, studies performed in cultured murine macrophages showed that DHA confers a protective effect against pro-inflammatory challenges, reducing drastically the magnitude of the response.

Furthermore, analysis of HSC phenotype revealed an important HSC deactivation in animals with ACLD treated with DHA, together with a decrease in extracellular matrix synthesis. Liver tissue fibrosis, however, failed to show a significant decrease in animals receiving DHA. This inconsistency between gene/protein expression determinations and liver histology analysis probably stems from the necessity of a longer period of time to allow extracellular matrix remodeling. Indeed, this is a complex process that requires the coordination of multiple enzymes to degrade the excessive matrix accumulation present in such advanced stages of liver disease [32,33], which suggests that a prolonged treatment with DHA could be more beneficial in reducing hepatic fibrosis. On the other hand, it is well known that liver vascular resistance is not only dependent on architectural distortion caused by fibrosis and parenchymal remodeling, but that an increased hepatic vascular tone contributes to further increase in intrahepatic resistance and portal pressure. This dynamic increase in liver vascular resistance has been well characterized and is mainly dependent on the existence of endothelial dysfunction at the intrahepatic circulation [3]. Intrahepatic endothelial dysfunction denotes a situation where the liver microcirculation losses its capacity to vasodilate in response to increased blood flow-related shear stress, is mainly determined by decreased nitric oxide (NO) bioavailability at the intrahepatic circulation, and aggravated by a shift in eicosanoid metabolism and increased endothelin production. In ACLD, intrahepatic endothelial dysfunction is multifactorial, and involves defective NO production due to decreased endothelial nitric oxide synthase (eNOS) activity, insufficient Krüppel-like factor 2 (KLF-2) expression, Rho A–Rho kinase dysregulation, increased asymmetric dimethylarginine (ADMA) production and, very importantly, NO scavenging by free oxygen radicals due to excessive oxidative stress [17]. Indeed, the importance of increased oxidative stress in liver endothelial dysfunction and increased hepatic vascular tone in ACLD has been highlighted by several studies from our group demonstrating the effectivity of pharmacological and genetic antioxidant and anti-inflammatory strategies improving portal hypertension and ACLD, at least at the bench side [34,35,36,37].

Considering the lack of effective and safe treatments for ACLD at the bedside [2,3], and the potential benefits described in the herein presented animal model of cirrhosis, we finally evaluated whether the improvement in rat HSC phenotype could be reproduced in human LX-2 HSC. Human cells treated with DHA showed a marked deactivation similar to what was observed in the animal model. These results, therefore, encourage the evaluation of this type of non-pharmacological therapeutic strategy as a new treatment for portal hypertension and cirrhosis.

In conclusion, the present study shows that ACLD pathophysiology involves a profound deregulation in the hepatic fatty acid composition. Treatment with a nutraceutical rich in DHA reestablished a healthy fatty acid profile. Moreover, cirrhotic animals that received DHA presented a moderate but significant improvement in portal hypertension. The mechanisms underlying this hemodynamic improvement included a reduction in oxidative stress and inflammation, as well as a marked HSC deactivation. Said deactivation was confirmed in human HSC, strengthening the interest of evaluating the use of DHA as a possible nutraceutical therapy for ACLD.

## Figures and Tables

**Table 1:** Effects of docosahexaenoic acid (DHA) administration on hepatic fatty acid composition in healthy and cirrhotic rats.

Fatty Acid	Healthy Vehicle	Healthy DHA	Cirrhotic Vehicle	Cirrhotic DHA
**SFA**				
MA (14:0)	0.39 ± 0.04	0.31 ± 0.03	0.43 ± 0.01	0.30 ± 0.02 **
PA (C16:0)	21.09 ± 0.31	21.48 ± 0.67	23.28 ± 0.41 ^###^	24.58 ± 0.39
SA (C18:0)	17.82 ± 0.26	17.40 ± 0.72	14.43 ± 0.88^###^	16.02 ± 0.54
**MUFA**				
POA (C16:1 *n*-7)	1.74 ± 0.17	1.68 ± 0.22	2.64 ± 0.30	1.74 ± 2.13 *
OA (C18:1 *n*-9)	8.40 ± 0.54	7.33 ± 0.40	15.70 ± 1.32 ^###^	8.87 ± 0.22 ***
VAC (C18:1 *n*-7)	3.77 ± 0.29	3.03 ± 0.10	3.36 ± 0.16	2.85 ± 0.16
EIA (C20:1 *n*-9)	0.13 ± 0.01	0.11 ± 0.01	0.12 ± 0.01	0.12 ± 0.01
NA (C24:1 *n*-9)	0.27 ± 0.02	0.24 ± 0.01	0.29 ± 0.03	0.34 ± 0.03
**PUFA**				
***n*** **-3 series**				
ALA (C18:3 *n*-3)	0.40 ± 0.04	0.43 ± 0.05	0.40 ± 0.03	0.25 ± 0.02 ***
ETE (C20:3 *n*-3)	0.30 ± 0,02	0.22 ± 0.02 *	0.28 ± 0.02	0.13 ± 0.02 **
EPA (C20:5 *n*-3)	0.18 ± 0.03	1.4 ± 0.26 **	0.11 ± 0.02	1.25 ± 0.08 ***
DPA (C22:5 *n*-3)	0.59 ± 0.03	1.41 ± 0.16 **	0.64 ± 0.03	1.42 ± 0.08 ***
DHA (C22:6 *n*-3)	5.85 ± 0.48	9.82 ± 0.52 **	1.97 ± 0.25 ^###^	11.70 ± 0.45 ***
***n*** **-6 series**				
LA (C18:2 *n*-6)	17.63 ± 0.54	18.37 ± 0.31	18.13 ± 0.42	17.32 ± 0.29
GLA (C18:3 *n*-6)	0.16 ± 0.01	0.11 ± 0.01 *	0.32 ± 0.02	0.08 ± 0.01 ***
HGLA (C20:3 *n*-6)	0.52 ± 0.05	0.84 ± 0.05 ***	0.,32 ± 0.02	0.82 ± 0.05 ***
AA (C20:4 *n*-6)	18.59 ± 0.32	14.76 ± 0.72 ***	14.86 ± 0.90 ^###^	10.25 ± 0.34 ***
DTA (C22:4 *n*-6)	0.49 ± 0.02	0.20 ± 0.02 ***	0.73 ± 0.07	0.21 ± 0.01 ***
DPA (C22:5 *n*-6)	0.26 ± 0.01	0.20 ± 0.02	1.07 ± 0.18	0.24 ± 0.02 ***
**Total SFA**	40.26 ± 0.54	40.11 ± 0.90	39.01 ± 0.53	42.07 ± 0.35 ***
**Total MUFA**	14.31 ± 0.93	12.34 ± 0.56	22.11 ± 1.55 ^###^	13.93 ± 0.36 ***
**Total PUFA**	45.44 ± 0.97	47.50 ± 0.74	38.88 ± 1.08 ^###^	43.99 ± 0.37 ***
**Total *n*-3 FA**	7.32 ± 0.54	12.94 ± 0.86 ***	3.11 ± 0.24 ^###^	14.74 ± 0.47 ***
**Total *n*-6 FA**	38.11 ± 0.76	34.55 ± 0.65 ***	35.77 ± 0.85	29.26 ± 0.33 ***

Results represent percentage (%) of total fatty acids and are expressed as mean ± S.E.M. *n* = 5 for healthy animals and *n* = 11 for cirrhotic animals. ^#^ <0.05; ^##^ <0.01; ^###^ <0.001 vs. healthy vehicle group and * <0.05; ** <0.01; *** <0.001 vs. corresponding group receiving vehicle. AA, arachidonic acid; ALA, alpha linolenic acid; DHA, docosahexaenoic acid; DPA, docosapentaenoic acid; DTA, docosatetraenoic acid; EIA, eicosenoic acid; EPA, eicosapentaenoic acid; ERA, erucic acid; ETE, eicosatrienoic acid; FA, fatty acid; GLA, gamma linoleic acid; HGLA, homogamma linolenic acid; LA, linoleic acid; MA, myristic acid; MUFA, monounsaturated fatty acid; NA, nervonic acid; OA, oleic acid; PA, palmitic acid; POA, palmitoleic acid; PUFA, polyunsaturated fatty acid; SA, stearic acid; SFA, saturated fatty acid; VAC, vaccenic acid.

**Table 2:** Effects of DHA administration on hepatic fatty acid ratios in healthy and cirrhotic rats.

Ratios	Healthy Vehicle	Healthy DHA	Cirrhotic Vehicle	Cirrhotic DHA
**SFA/MUFA**	2.86 ± 0.44	3.27 ± 0.48	1.81 ± 0.34 ^###^	3.04 ± 0.30 ***
**MUFA/PUFA**	0.32 ± 0.06	0.26 ± 0.03	0.58 ± 0.13 ^##^	0.32 ± 0.03 ***
**ω-6/ω-3**	5.32 ± 0.88	2.72 ± 0.46 **	11.72 ± 1.56 ^###^	2.01 ± 0.26 ***
**ω-3 INDEX** **(DHA + EPA)**	6.03 ± 1.09	11.15 ± 1.64 ***	2.08 ± 0.57 ^###^	12.95 ± 1.49 ***
**AA/EPA**	108.3 ± 25.2	12.74 ± 5.69 ***	156.0 ± 24.2 ^###^	8.65 ± 2.35 ***
**AA/DHA**	3.26 ± 0.58	1.50 ± 0.33 ***	7.87 ± 1.41 ^###^	0.90 ± 0.18 ***
**Δ5** **(C20:4 *n*-6/C20:3 *n*-6)**	36.99 ± 3.95	17.39 ± 1.152 ***	47.55 ± 2.12	12.09 ± 0.85 ***
**Δ6** **(C20:3 *n*-6/C18:2 *n*-6)**	2.99 ± 0.31	4.58 ± 0.27	1.76 ± 0.10	4.76 ± 0.31 ***
**SCD1 INDEX** **(C16:1 *n*-7/C18:1 *n*-9)**	20.72 ± 1.81	22.98 ± 2.79	16.80 ± 1.33	19.74 ± 1.51
**SCD16 INDEX** **(C16:1 *n*-7/C16:0)**	8.24 ± 0.81	7.73 ± 0.78	11.30 ± 1.17	7.08 ± 0.49
**SCD18 INDEX** **(C18:1 *n*-9/C18:0)**	47.32 ± 3.70	42.72 ± 3.76	111.6 ± 14.1 ^###^	56.02 ± 2.63 ***
**C18:2 *n*-6/C20:4 *n*-6**	0.95 ± 0.02	1.29 ± 0.08	1.24 ± 0.09	1.71 ± 0.08
**C18:0/C16:0**	0.85 ± 0.01	0.81 ± 0.04	0.62 ± 0.05 ^#^	0.66 ± 0.03
**C24:0/C22:0**	2.82 ± 0.12	3.39 ± 0.20	2.53 ± 0.22	3.01 ± 0.12
**C24:0/C20:0**	7.77 ± 0.73	7.28 ± 0.58	3.90 ± 0.35 ^#^	5.73 ± 0.35
**C18:1 *n*-7/C16:1 *n*-7**	2.22 ± 0,18	1.92 ± 0.23	1.34 ± 0.17	1.67 ± 0.11 **

Results are expressed as mean ± S.E.M. *n* = 5 for healthy animals and *n* = 11 for cirrhotic animals. ^#^ <0.05; ^##^ <0.01; ^##^ <0.001 vs. healthy vehicle group and * <0.05; ** <0.01; *** <0.001 vs. corresponding group receiving vehicle. AA, arachidonic acid; DHA, docosahexaenoic acid; EPA, eicosapentaenoic acid; PUFA, polyunsaturated fatty acid; MUFA, monounsaturated fatty acid; SFA, saturated fatty acid.

**Table 3:** Effects of DHA administration on hepatic and systemic hemodynamics and serum markers in a rat model of advanced chronic liver disease (ACLD).

Parameter	Vehicle	DHA	*p* Value
**PP (mmHg)**	13.91 ± 0.60	12.05 ± 0.57	0.03
**PBF (mL·min^−1^)**	15.66 ± 2.54	18.21 ± 2.31	>0.20
**IHVR (mmHg·min·mL^−1^)**	1.05 ± 0.15	0.79 ± 0.11	0.16
**SMABF (mL·min^−1^)**	8.37 ± 0.91	10.03 ± 1.45	>0.20
**MAP (mmHg)**	116.23 ± 5.40	99.92 ± 6.25	0.1
**HR (beats·min^−1^)**	415 ± 13	383 ± 17	0.15
**AST (U/L)**	155.8 ± 17.08	115.8 ± 9.05	0.04
**Albumin (U/L)**	31.2 ± 0.32	30.27 ± 0.53	>0.20

Results are expressed as mean ± S.E.M. *n* = 11 and *n* = 14, respectively, for vehicle and DHA. PP, portal pressure; MAP, mean arterial pressure; PBF, portal blood flow; IHVR, intrahepatic vascular resistance; SMABF, superior mesenteric arterial blood flow; HR, heart rate; AST, aspartate aminotransferase.

## Supplementary Materials

The following are available online at https://www.mdpi.com/2072-6643/11/10/2358/s1, Table S1: List of primer sequences for quantitative PCR studies, Table S2: List of antibodies used for western blot studies, Table S3: Effects of DHA administration on body and organ weight in a rat model of ACLD.
