# Supplementary material for: A Nutraceutical Rich in Docosahexaenoic Acid Improves Portal Hypertension in a Preclinical Model of Advanced Chronic Liver Disease
Source: Nutrients. 2019 Oct 3;11(10):2358. doi: 10.3390/nu11102358 (PMC6835927; doi:10.3390/nu11102358)
Supplement: Supplementary file 1 [file nutrients-11-02358-s001.pdf]

Supplementary materials: A nutraceutical rich in docosahexaenoic acid improves portal hypertension in a preclinical model of advanced chronic liver disease

Zoe Boyer-Diaz, Joan Carles Domingo, Estefanía de Gregorio, Nicolò Manicardi, Peio Aristu-Zabalza, Begoña Cordobilla, Laia Abad-Jorda, Martí Ortega-Ribera, Anabel Fernández-Iglesias, Montserrat Marí, Jaime Bosch and Jordi Gracia-Sancho

Supplementary Table S1. List of primer sequences for quantitative PCR studies.

Rattus norvegicus genes	Forward (5'→3')	Reverse (3'→5')	Product size (bp)
**IL-1β**	GCTTCCTTGTGCAAGTGTCT	TCTGGACAGCCCAAGTCAAG	160
**IL-6**	TCCGGAGAGGAGACTTCACA	GCCATTGCACAACTCTTTTCT	128
**IL-10**	CCTGGTAGAAGTGATGCCCC	AGACACCTTTGTCTTGGAGCTTAT	194
**α-SMA**	CTCATGCCATCATGCGTCTG	CACGCTCAGCAGTAGTCACG	105
**collagen-1α1**	GTACATCAGCCCAAACCCCA	TCGCTTCCATACTCGAACTGG	87
**gapdh**	GGCATCGTGGAAGGGCTCAT	AGGGATGATGTTCTGGGCTGC	120
***Mus musculus* genes**	**Forward (5'→3')**	**Reverse (3'→5')**	**Product size (bp)**
**IL-6**	CCGGAGAGGAGACTTCACAG	CAGAATTGCCATTGCACAAC	136
**tnfα**	CTGAACTTCGGGGTGATCGGT	ACGTGGGCTACAGGCTTGTCA	134
**Arg1**	TCGGAACTCAACGGGAGGGT	TGCTTTGCTGTGATGCCCCA	108
**β-actin**	GACGGCCAGGTCATCACTAT	CGGATGTCAACGTCACACTT	140

Supplementary Table S2. List of antibodies used for western blot studies.

Primary antibodies	Supplier	Species	Type	Dilution	Reference
**α-SMA**	Sigma	Mouse	Monoclonal	1:1000	A2547
**Collagen-1α1**	Cell Signaling	Rabbit	Polyclonal	1:1000	84336S
**IL-1β**	Abcam	Rabbit	Polyclonal	1:1000	ab9722
**IL-6**	Abcam	Mouse	Monoclonal	1:1000	ab9324
**TGFβ**	Santa Cruz	Rabbit	Polyclonal	1:1000	SC-398
**PDGFRβ**	Santa Cruz	Goat	Polyclonal	1:1000	SC-1627
**p65-NFκB**	Santa Cruz	Rabbit	Polyclonal	1:200	SC-372
**GAPDH**	Santa Cruz	Mouse	Monoclonal	1:1000	SC-32233
**Lamin A/C**	Santa Cruz	Goat	Polyclonal	1:200	SC-6215
**Secondary antibodies**	**Supplier**	**Species**	**Type**	**Dilution**	**Reference**
**Anti-Mouse IgG (HRP conjugate)**	Sigma	Rabbit	Polyclonal	1:10000	A9044
**Anti-Rabbit IgG (HRP conjugate)**	Enzo Life Sciences	Goat	Polyclonal	1:10000	ADI-SAB-300-J
**Anti-Goat IgG (HRP conjugate)**	Santa Cruz	Mouse	Monoclonal	1:10000	SC-2354

Supplementary Table S3. Effects of DHA administration on body and organ weight in a rat model of ACLD.

Parameter	Vehicle	DHA	p value
**Body weight (g)**	475 ± 11	506 ± 16	0.13
**Liver weight (g)**	15.19 ± 0.97	15.26 ± 0.90	> 0.20
**Spleen weight (g)**	1.39 ± 0.08	1.32 ± 0.09	> 0.20

Results are expressed as mean ± S.E.M. n=11 and n=14, respectively, for vehicle and DHA.
